# Gain-Enhanced Metamaterial Absorber-Loaded Monopole Antenna for Reduced Radar Cross-Section and Back Radiation

**DOI:** 10.3390/ma13051247

**Published:** 2020-03-10

**Authors:** Heijun Jeong, Yeonju Kim, Manos M. Tentzeris, Sungjoon Lim

**Affiliations:** 1 School of Electrical and Electronics Engineering, College of Engineering, Chung-Ang University, Seoul 06974, Korea; jhijun000015@gmail.com (H.J.); yjkim-430@hanmail.net (Y.K.); 2 School of Electrical and Computer Engineering, College of Engineering, Georgia Institute of Technology, Atlanta, GA 30332, USA

**Keywords:** high gain monopole antenna, metamaterial absorber, back radiation reduction, RCS reduction

## Abstract

This paper proposes a gain-enhanced metamaterial (MM) absorber-loaded monopole antenna that reduces both radar cross-section and back radiation. To demonstrate the proposed idea, we designed a wire monopole antenna and an MM absorber. The MM absorber comprised lumped elements of subwavelength unit cells and achieved 90% absorbance bandwidth from 2.42–2.65 GHz. For low-profile configurations, the MM absorber was loaded parallel to and 10 mm from the monopole antenna, corresponding to 0.09 λ_0_ at 2.7 GHz. The monopole antenna resonated at 2.7 GHz with a 3.71 dBi peak gain and 2.65 GHz and 6.46 dBi peak gain, before and after loading the MM absorber, respectively. Therefore, including the MM absorber increased peak gain by 2.7 dB and reduced back radiation by 15 dB. The proposed antenna radar cross-section was reduced by 2 dB compared with a monopole antenna with an artificial magnetic conductor.

## 1. Introduction

Metamaterials (MMs) are periodic structures with a nominally infinite number of artificial structures [1] designed to control permittivity and permeability. Hence, MMs have been employed for many electromagnetic applications, such as terahertz devices [2], frequency selective surfaces [3], and super lenses [4], and particularly for antenna applications, such as electrically small [5], high gain [6], and beam-scanning [7] antennas.

Antenna gain can be increased with suitable reflectors, but the reflector must be placed a quarter-wavelength (*λ*/4) from the antenna to avoid cancelling the original and image currents, which is not ideal for low-profile antennas. To create a high-gain antenna with a low profile, we replaced the conventional reflector (similar to a perfect electric conductor (PEC)) with MMs, forming an artificial magnetic conductor (AMC) [8], or high impedance surface [9], due to its similar reflection coefficient as perfect magnetic conductors at resonance frequency [10,11,12]. High-gain antennas can be created using an AMC as the reflective ground [13,14,15], or superstrate [16,17,18]. Despite their high-gain and low-profile configuration, the antenna radar cross-section (RCS) with AMC is similar to that of conductive plates, due to the reflected wave from the AMC.

At the same time, MM absorbers have been widely used to reduce RCS, as MM absorbers can absorb electromagnetic waves, with the result that many MM absorber-based antennas are used as radar-absorbing materials for radar applications [19,20,21]. However, most radar-absorbing materials focus on RCS reduction, rather than enhancing gain or reducing back radiation.

This study proposes adding an MM absorber to a monopole antenna to reduce RCS while simultaneously enhancing gain, reducing back radiation, and offering beam reflection. The MM absorber incorporates lumped elements for subwavelength unit cells and achieves a low profile by loading parallel to the monopole antenna at a minimum distance. Proposed antenna performance was numerically and experimentally compared with monopole, monopole-with-PEC, and monopole-with-AMC antennas, exhibiting higher gain, lower back radiation, higher front-to-back ratio (FBR), and lower RCS.

## 2. Proposed Antenna Design

**Figure 1:**
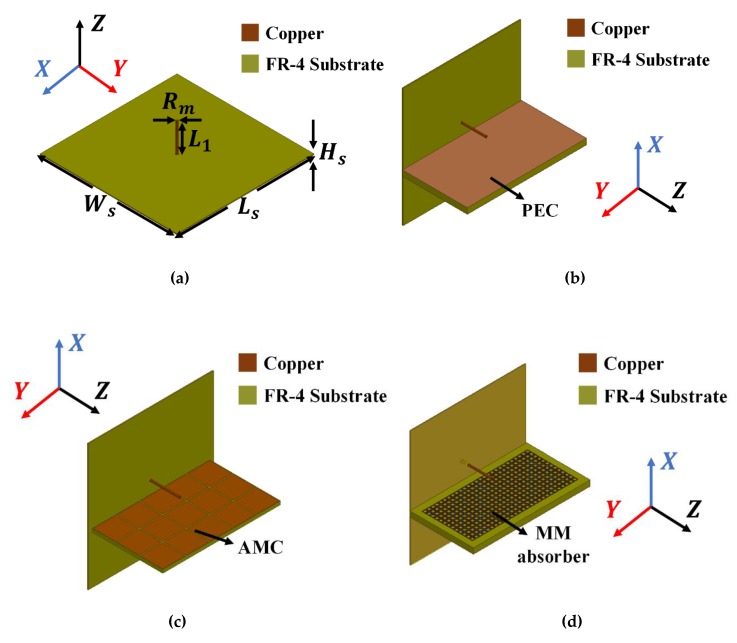
Monopole antennas: (**a**) reference antenna 1: bare, (**b**) reference antenna 2: perfect electric conductor (PEC), (**c**) reference antenna 3: artificial magnetic conductor (AMC), and (**d**) proposed antenna: metamaterial (MM) absorber.

We simulated the proposed antenna for electromagnetic analysis using ANSYS High Frequency Structure Simulator (HFSS) software (Version 17.2, ANSYS, Canonsburg, PA, USA). Figure 1 shows the proposed and three reference antennas, where the proposed antenna comprised a monopole antenna and MM absorber (Figure 1d). A quarter-wavelength vertical monopole antenna was designed for all antennas with 2.7 GHz resonance and geometrical parameters as shown in Table 1. We assumed copper conductivity = 5.8 × 10^7^ S/m, and FR-4 substrate dielectric constant = 3.9 and loss tangent = 0.02. An air radiation box was used for the radiation boundary with dimension 158.5 × 110.8 × 157 mm^3^. A sub-miniature version A (SMA) connector was included to provide excitation, and the wave port was assigned to the coaxial transmission line.

### 2.1. Metamaterial Absorber Design

**Figure 2:**
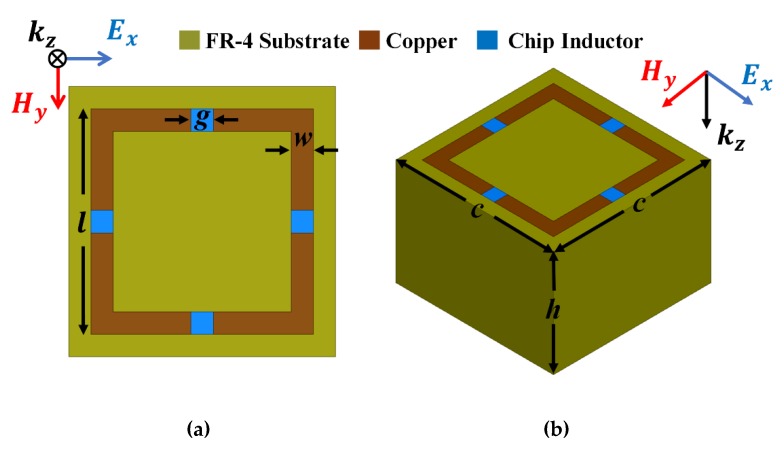
Proposed MM absorber unit cell: (**a**) top and (**b**) perspective view.

Figure 2 shows the MM absorber unit cell that will be loaded on the monopole antenna in parallel. High MM absorptivity can be achieved by minimizing reflection Γ(ω) and transmission T(ω) coefficient, with total absorptivity

(1) A(ω)=1−Γ(ω)−T(ω)

T(ω) can be made zero by fully covering the MM absorber bottom plane; and under normal incidence, Γ(ω) can made zero by matching MM impedance (Z_M_) of that for free space (Z_0_ = 377 Ω), since

(2) $$\Gamma (\omega )=\frac{Z_{0}-Z_{M}}{Z_{0}+Z_{M}}$$

The MM unit cell was designed based on inductive-capacitive (LC) resonance. Resonance frequency depends on 1/√LC, hence decreases with increasing inductance (L). Thus, four 17 nH chip inductors were loaded on square split-ring resonator gaps to miniaturize the unit cell [22]. The conductive pattern was designed on FR-4 substrate, with dielectric constant (ε_r_) = 3.9; loss tangent tan δ = 0.02; and dimensions l = 3.3 mm, g = 0.5 mm, w = 0.5 mm, c = 4 mm, and h =5.6 mm.

**Figure 3:**
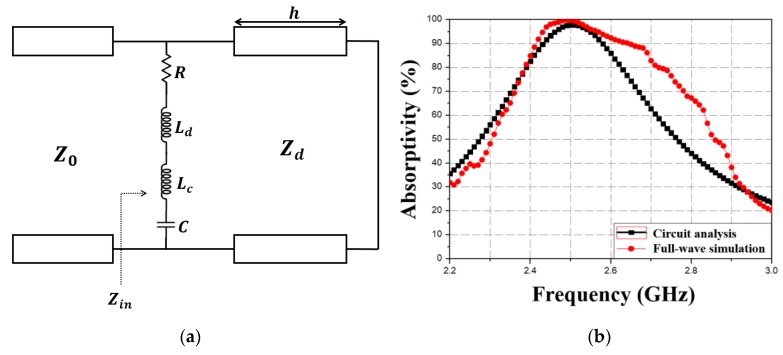
(**a**) Proposed MM absorber equivalent circuit model and (**b**) corresponding simulated absorptivity using full-wave simulation.

Figure 3 shows MM absorber equivalent circuit and simulated absorptivity, where Z_0_ and Z_d_ represent free space and dielectric substrate impedance, respectively. Top conductive patterns can be represented as series R, L, and C; where R represents conductive pattern resistance, L represents the sum of inductance due to conductive patterns (L_d_) and chip inductors (L_c_); and C is capacitance due to the gap between unit cells. Thus, resonance frequency can be expressed as

(3) $$f_{r}=\frac{1}{2\pi \sqrt{(L_{c}+L_{d})\times C}}=\frac{1}{2\pi \sqrt{L_{eff}\times C}}$$

Circuit parameter values for the equivalent circuit (Figure 3a) were extracted using the Keysight Advanced Design System (ADS) simulator, with R = 33.6 Ω, L_d_ = 8.1 nH, L_c_ = 17 nH, and C = 0.15 pF. The reflection coefficient and absorptivity were subsequently calculated from Z_in_. Figure 3b compares calculated absorptivity from the equivalent circuit model with simulated absorptivity from ANSYS HFSS. The slight difference between calculated and simulated 90% absorptivity ranges (2.43–2.59 and 2.42–2.65 GHz, respectively) is due to the circuit parameters being derived for 2.5 GHz.

**Figure 4:**
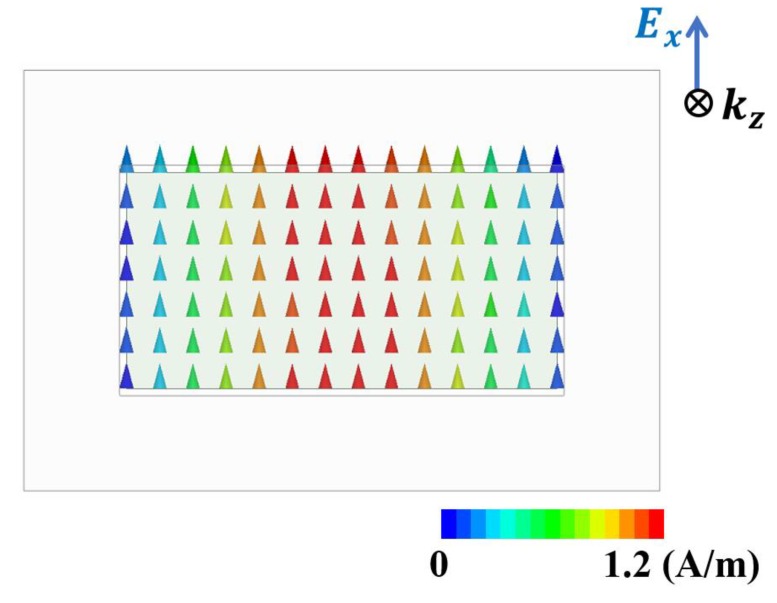
Simulated electric-field vector at the rectangular waveguide port.

The proposed MM absorber is operating in the waveguide, which is somewhat different than free space. The rectangular waveguide supports a transverse electric (TE) mode at 2.65 GHz, propagating in the z-direction. Therefore, the magnetic field is polarized along y- and z-directions whereas the electric field is polarized along the x-direction, as shown in Figure 4. Consequently, the proposed MM absorber incident angle-insensitive characteristics were not characterized because the incident angle cannot be changed under the waveguide setup.

### 2.2. Proposed Antenna with Metamaterial Absorber

**Figure 5:**
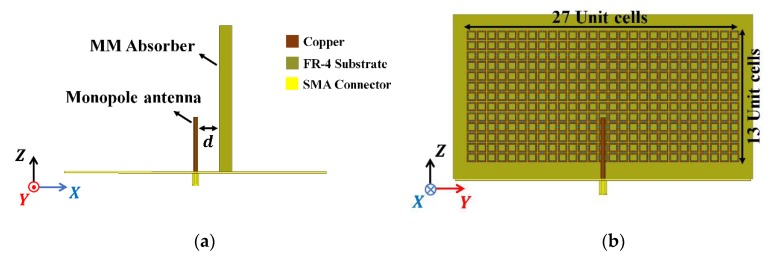
Proposed antenna: (**a**) side and (**b**) top view.

Figure 5a shows the proposed antenna comprising the monopole antenna-parallel MM absorber. The MM absorber included 13 × 27 unit cells with overall dimensions 116.5 × 62.5 × 5.6 mm^3^ (Figure 5b).

**Figure 6:**
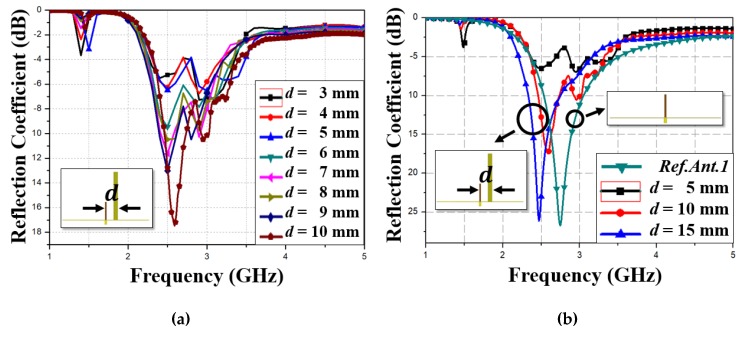

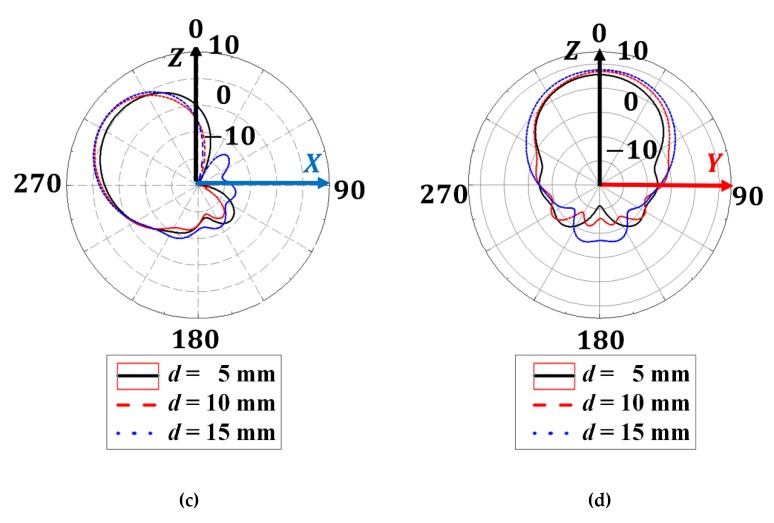
Simulated reflection coefficients for (**a**) proposed antenna and (**b**) reference antenna 1 (Ref. Ant. 1) with respect to antenna and MM separation (d); simulated two-dimensional radiation patterns at 2.65 GHz on (**c**) XZ and (**d**) YZ planes for different d.

Figure 6a shows the simulated antenna reflection coefficient for MM absorber separation d = 3–10 mm, in 1 mm steps. Antenna impedance is not matched below d = 6 mm due to coupling between the antenna and absorber, but reflection coefficient = −11, −10, −13, and −17 dB for d = 7, 8, 9, and 10 mm, respectively, at 2.65 GHz. Therefore, the antenna could be as close as 7 mm from the MM absorber. However, considering fabrication we wanted reflection coefficient magnitude > 15 dB, hence we set d = 10 mm. 

To achieve maximal directivity, antenna S-parameters and radiation patterns were simulated for different d. Figure 6b shows monopole (Ref. Ant. 1) and proposed antenna simulated reflection coefficients for d = 5, 10, and 15 mm. Impedance was not matched when d = 5 mm, due to strong coupling between the antenna and absorber; whereas reflection coefficient = −17 and −26 dB at 2.65 and 2.49 GHz when d = 10 and 15 mm, with resonance at 2.65 and 2.49 GHz (respectively. This reduced resonance (0.05 and 0.21 GHz reduction, respectively) was due to coupling between the antenna and the MM absorber. 

Figure 6c,d shows corresponding two-dimensional radiation patterns on XY and YZ planes for the proposed antenna at d = 5, 10, and 15 mm, with peak gain 5.34, 6.46, and 6.67 dBi at 2.65 GHz, respectively. A minimum d is desirable for a low profile, hence we selected d = 10 mm to simultaneously provide low profile and high gain, i.e., 0.09 λ_0_ at 2.65 GHz.

**Figure 7:**
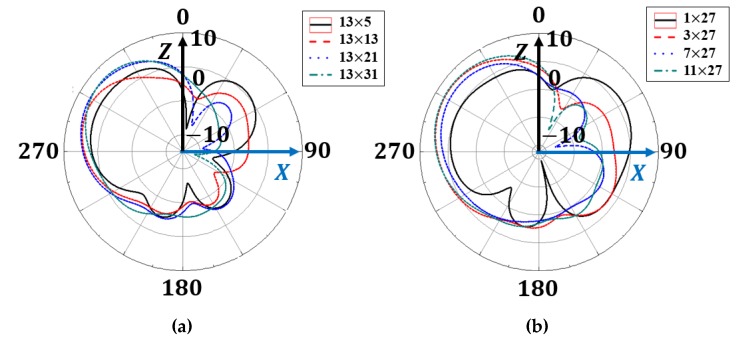
Simulated radiation patterns for different metamaterial absorber (MMA) array (**a**) rows and (**b**) columns.

Figure 7 shows simulated radiation patterns for different MM array sizes. Front radiation increases, whereas back radiation decreases, with increasing MM array row length (Figure 7a), with the level of change decreasing with row length > 21. Similarly, front radiation increases and back radiation decreases with increasing MM array column length, although the rate of change reduces with number of rows >7.

**Figure 8:**
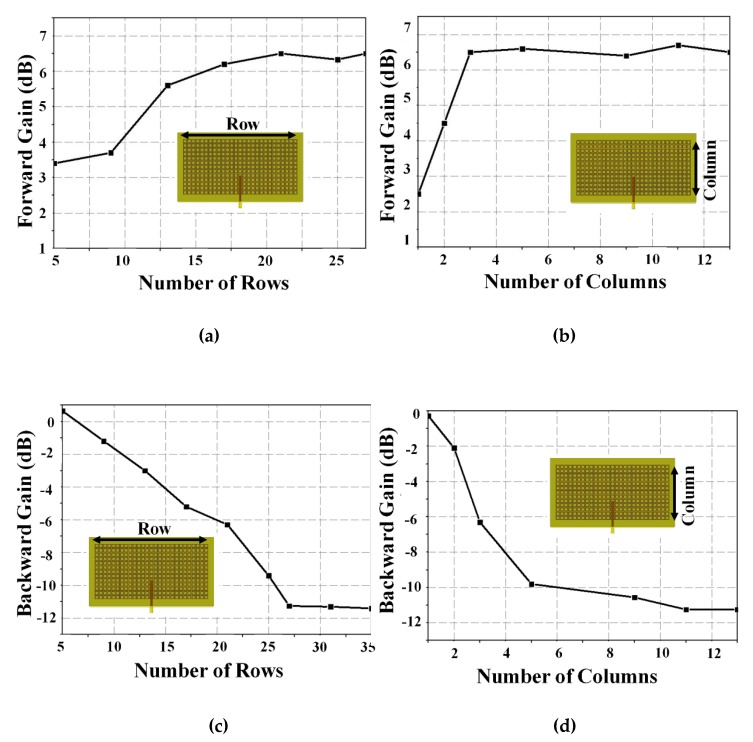
Simulated forward gain (θ = 0°) for the proposed antenna at 2.65 GHz with different (**a**) rows and (**b**) columns of unit cells. Simulated backward gain (θ = 180°) for the proposed antenna at 2.65 GHz with different (**c**) rows and (**d**) columns of unit cell.

We then simulated peak gain for different numbers of unit cells in the MM absorber to identify optimal absorber overall dimensions. Figure 8a shows the proposed antenna peak gain at θ = 0° for 5–30 unit cell rows. Peak gain at 2.65 GHz increased with increasing rows, saturating at approximately 21 rows. Figure 8b shows peak gain at 2.7 GHz for 1–13 unit cell columns. Peak gain was not affected for columns > 3. Figure 8c shows back radiation (i.e., θ = 180°) for 5–35 unit cell rows. Back radiation at 2.65 GHz reduced as the number of rows increased, saturating at approximately 27 rows. Figure 8d shows back radiation for 1–13 unit cell columns. Back radiation at 2.65 GHz decreased as the number of columns increased, saturating after 7 columns. Thus, for best performance of the proposed antenna, the MM absorber should include more than 7 × 27 unit cells.

### 2.3. Comparison of Proposed Antenna with Reference Antennas

**Figure 9:**
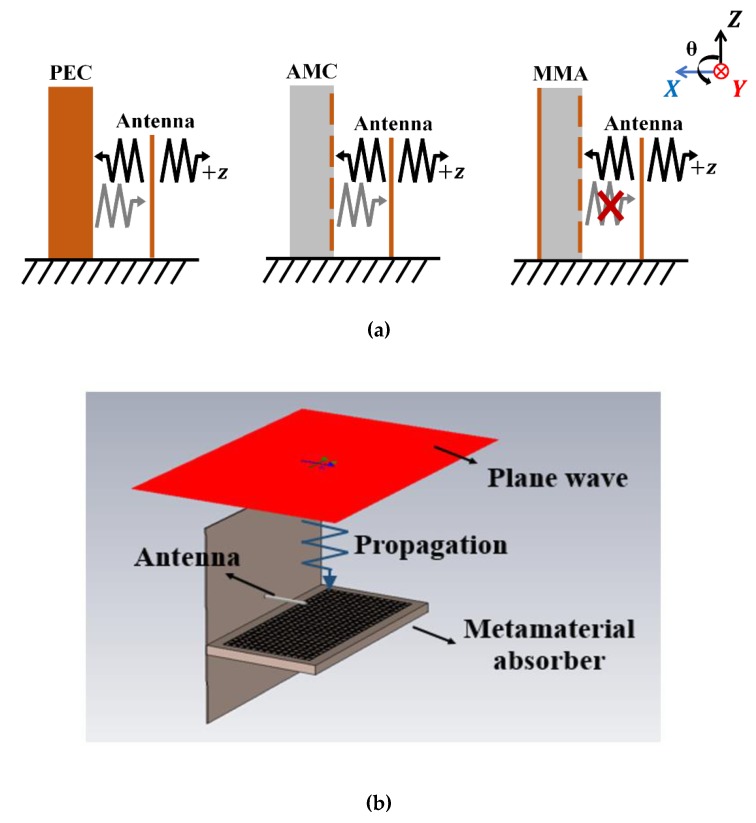

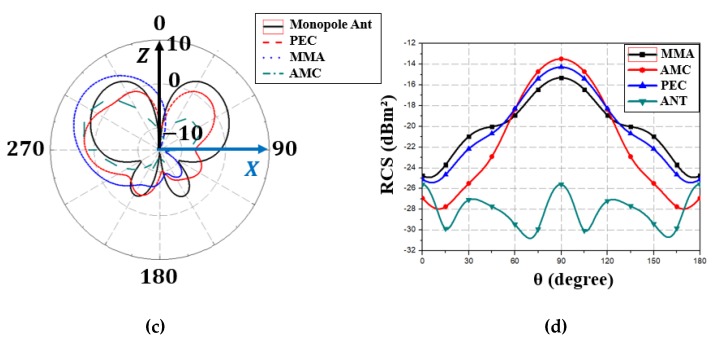
(**a**) Monopole antenna and reflection radiation patterns from perfect electric conductor (PEC), artificial magnetic conductor (AMC), and MM absorbers. (**b**) Simulation setup for RCS analysis in the Computer Simulation Technology (CST) Microwave. (**c**) Simulated two-dimensional radiation patterns for the proposed and reference antennas at 2.65 GHz. (**d**) Simulated radar cross-section (RCS) for the proposed and reference 1, 2, and 3 antennas at 2.65 GHz.

Proposed antenna performance was compared with the reference antennas (see Figure 1). Figure 9a shows that the monopole antenna must be placed at λ/4 from the PEC to avoid cancelling the radiated wave from the monopole antenna and reflected wave from the PEC. However, the proposed monopole antenna can be placed much closer than λ/4 because the AMC reflected wave is in phase with the radiated wave from the monopole antenna. In contrast, the proposed antenna exhibits no reflected wave from the MM absorber. Figure 9c shows two-dimensional radiation patterns for the proposed and reference antennas on the XZ plane at 2.65 GHz, and Figure 9d shows simulated RCS for the proposed and reference antennas. Figure 9b shows simulated RCS analysis using the computer simulation technology (CST) microwave studio. An incident plane wave was simulated toward the MM absorber and reflected waves were calculated as the RCS wave. 

Figure 9d shows that reference antenna 1 exhibited the lowest RCS because there were no metallic structures around the antenna. However, reference antenna 1 is omnidirectional and PEC or AMC reflectors are generally used to increase directivity. RCS is increased when PEC or AMC are loaded on the antenna due to their metallic structures. On the other hand, the MM absorber can reduce RCS because the incident electromagnetic wave is absorbed. 

Table 2 summarizes antenna performances and also shows FBR calculated from (gain at θ = 0°)/(gain at θ = 180°). Reference antenna 1 peak gain increased by 2.36 dB from 1.35 dBi to 3.71 dBi when the bottom ground was placed. When the PEC structure was arranged parallel to the monopole antenna, reference antenna 2 peak gain decreases by 0.41 dB from 3.71 dBi to 3.3 dBi, because reference antenna 1 distance (d) is not optimized. When the distance is optimized, reference antenna 2 peak gain increased by 4.3 dB from 3.3 dBi to 7.6 dBi. When AMC was loaded, reference antenna 3 peak gain increased by 2.54 dB from 3.71 dBi to 6.25 dBi. Thus, AMC acts as a reflector to enhance the gain. Finally, the proposed antenna peak gain increased by 2.75 dB from 3.71 dBi to 6.46 dBi. Thus, the proposed antenna achieved high peak gain, lowest RCS, and highest FBR.

**Figure 10:**
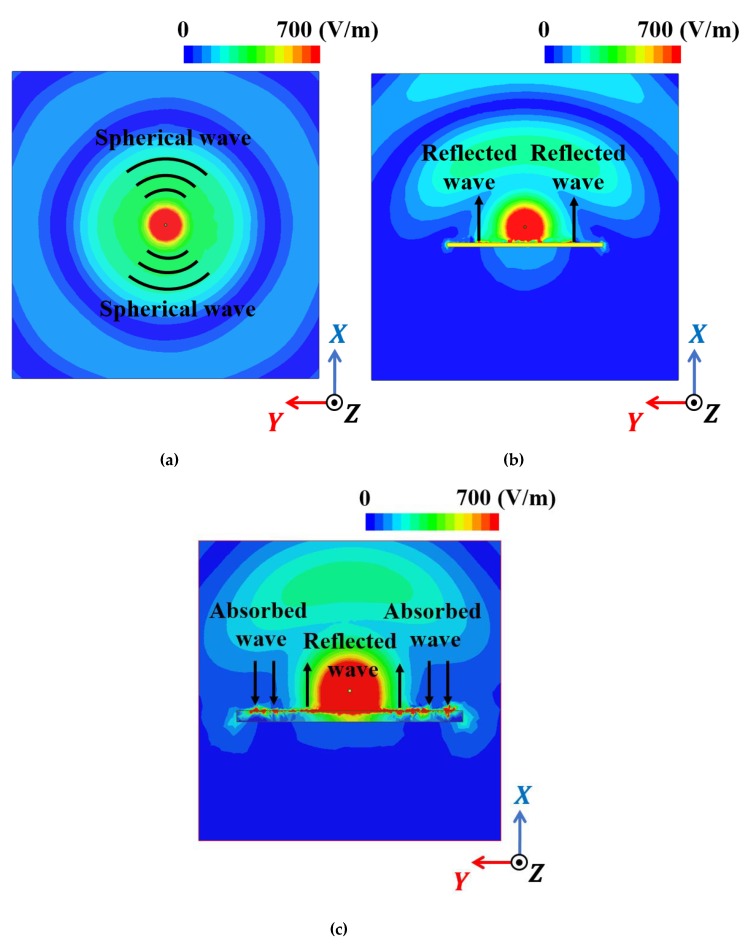
Simulated electric field distribution top views for (**a**) reference 1 (monopole), (**b**) reference 3 (monopole antenna with AMC), and (**c**) proposed (monopole with MM) antenna.

Figure 10 shows the total electric field magnitude for the proposed antenna, and reference antennas 1 and 3 to investigate the working mechanism. Figure 10a shows the spherical EM wave is radiating from reference antenna 1, Figure 10b shows that the reflected EM wave from AMC increased reference antenna 3 directivity, and Figure 10c shows the partially absorbed EM wave absorbed by the MM absorber while the remainder of the EM wave is reflected. In particular, the partially reflected EM wave increases the proposed antenna directivity. The proposed antenna EM wave is close to hemispherical, whereas that from reference antenna 3 was close to spherical.

To experimentally demonstrate the proposed antenna performance, we fabricated the wire monopole antenna, MM absorber, and reference antennas 1 and 2. The monopole antenna and MM absorber ground planes were built on FR4 substrate using printed circuit board etching, and the 1608 chip inductors were mounted for the MM absorber. 

**Figure 11:**
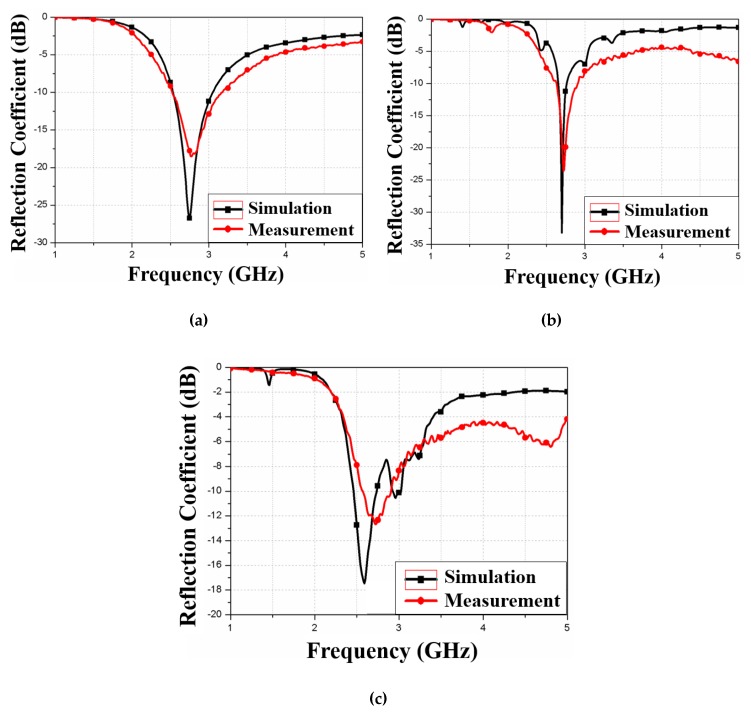
Simulated and measured reflection coefficients for (**a**) reference antenna 1, (**b**) reference antenna 2, and (**c**) proposed antenna.

Figure 11 shows the simulated and measured reflection coefficient of these three antennas. Figure 11a shows the simulated and measured reflection coefficients for reference antenna 1, with measured reflection coefficient = −18 dB at 2.7 GHz, compared with −27 dB at 2.7 GHz for the simulation. Figure 11b shows simulated and measured reflection coefficients for reference antenna 2, with measured reflection coefficient = −23 dB at 2.7 GHz compared with −33 dB at 2.7 GHz for the simulation. Finally, Figure 11c shows simulated and measured reflection coefficients for the proposed antenna, with measured and simulated reflection coefficients = −13 and −17 dB at 2.7 GHz, respectively. Thus, the simulated and measured results show good agreement for all three antennas.

**Figure 12:**
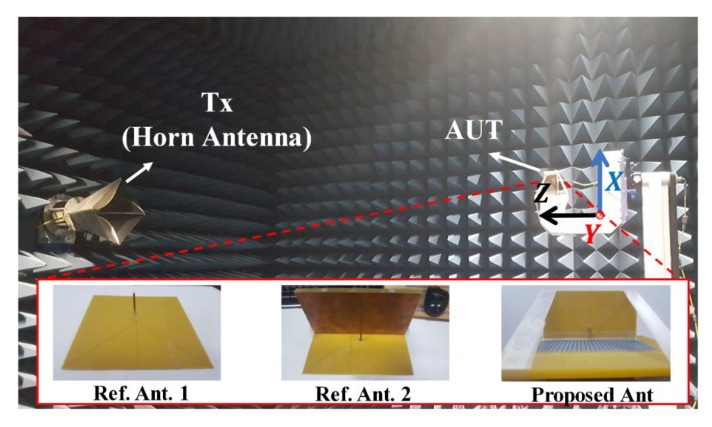
Radiation pattern measurement setup and fabricated antennas.

**Figure 13:**
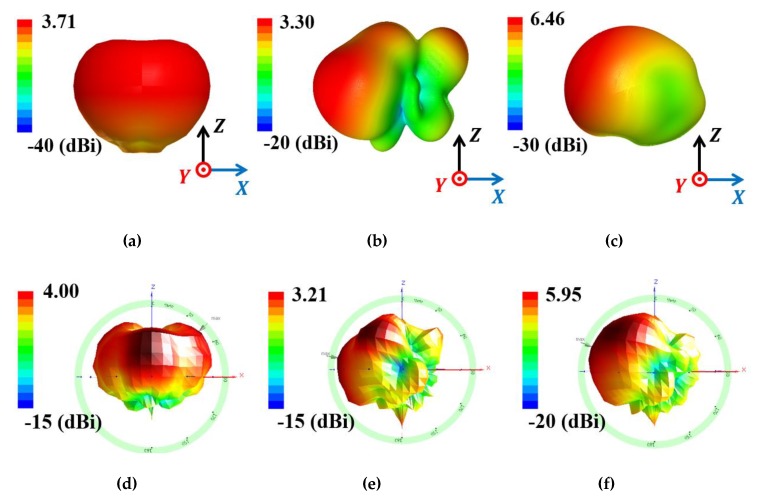
Simulated and measured 3D radiation patterns at 2.65 GHz for (**a**) and (**d**) reference antenna 1, (**b**) and (**e**) reference antenna 2, and (**c**) and (**f**) proposed antenna.

Figure 12 shows the setup to measure radiation patterns for the proposed and reference antennas, and Figure 13 compares simulated and measured 3D radiation patterns at 2.7 GHz. Reference antenna 1 shows monopolar radiation pattern with simulated and measured peak gain = 3.71 and 4 dBi (Figure 13a,d, respectively). Figure 13b,e shows the directional radiation pattern for reference antenna 2, with simulated and measured peak gain = 3.3 and 3.21 dBi, respectively. Figure 13c,f shows the proposed antenna simulated and measured peak gain = 6.46 and 5.95 dBi, respectively. 

Thus, the measured peak gain increased by 19.5 dB by loading the MM absorber, compared to the monopole antenna; back radiation was also reduced by 15 dB. Table 3 compares the proposed antenna performance with that of other gain-enhanced antennas using MM-based reflectors. The proposed antenna shows a high FBR with a low-profile configuration. Although RCSs were not compared, a lower RCS is expected from the proposed antenna than other antennas due to the MM absorber.

**Figure 14:**
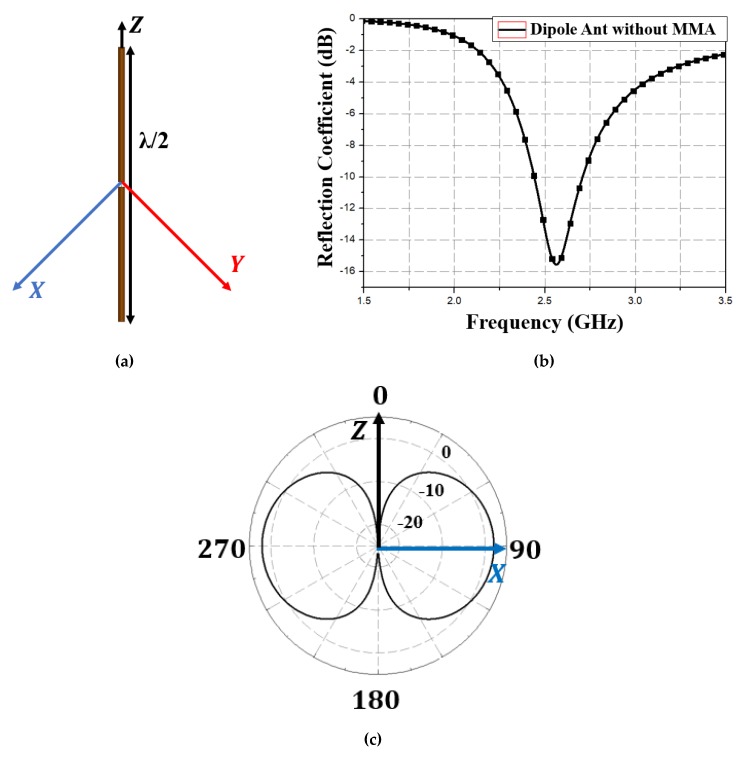
Dipole antenna (**a**) geometry (**b**) simulated reflection coefficient, and (**c**) two-dimensional radiation pattern at *ϕ* = 0 and 90°.

The proposed concept is also applicable to dipole antennas. Figure 14 shows a typical half-wavelength dipole antenna and corresponding simulated results. Figure 14a shows the antenna resonance at 2.65 GHz; Figure 14c shows the corresponding radiation pattern on the XZ plane. 

**Figure 15:**
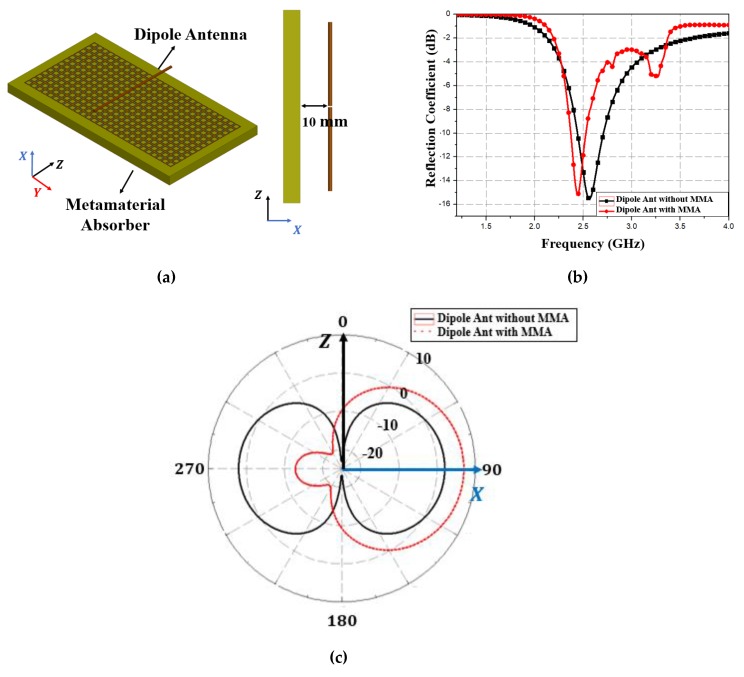
Proposed MM absorber loaded dipole antenna (**a**) geometry (**b**) simulated reflection coefficient compared with bare dipole antenna, and (**c**) two-dimensional radiation patterns at *ϕ* = 0 and 90°.

Figure 15a shows that loading the half-wavelength dipole antenna on the MM absorber at a 10 mm distance slightly reduced resonance frequency from 2.65 to 2.57 GHz (0.08 GHz reduction), due to coupling between the dipole antenna and MM absorber, as shown in Figure 15b. Figure 15c shows dipole antenna radiation patterns with and without the MM absorber. Peak gain on the XZ and XY planes increased by 5 dBi from 2 to 7 dBi, and back radiation reduced from 2 to −12 dB due to the MM absorber. Thus, the proposed concept applies equally to a dipole antenna.

## 3. Conclusions

This paper proposed a high-gain MM absorber-loaded monopole antenna to reduce RCS and back radiation. A low profile was achieved by setting the distance between the MM absorber and the monopole antenna at 10 mm (0.09 λ_0_ at 2.7 GHz). The proposed antenna performance was numerically compared with a bare monopole antenna and the same antenna on PEC and AMC. The proposed antenna achieved a 2.75 dB increase in peak gain compared with the bare antenna, and 2 dB lower RCS than the monopole antenna with AMC. Experimental results showed the proposed antenna achieved a peak gain = 5.95 dBi and FBR = 16 dB at 2.65 GHz. Thus, the proposed approach would be suitable for applications requiring low RCS and back radiation, such as stealth technology.

## Figures and Tables

**Table 1:** Proposed monopole antenna parameters.

Parameter	Value (mm)	Description
$L_{1}$	25	Wire monopole length
$R_{m}$	2	Wire monopole diameter
$W_{s}$	117	FR-4 substrate width
$L_{s}$	117	FR-4 substrate length
$H_{s}$	0.8	FR-4 substrate thickness

**Table 2:** Simulation performance metrics for proposed and reference antennas.

Antenna	Gain at θ = 0° (dB)	Gain at θ = 180° (dB)	RCS (dBm^2^)	FBR (dB)	d (mm)
Bare monopole antenna (without bottom ground)	1.35	1.35	N/A	0	N/A
Reference antenna 1	3.71	3.71	N/A	0	10
Reference antenna 2	3.3	0.9	−13.8	2.4	10
Reference antenna 3	7.6	−1.2	−13.8	8.8	22
Reference antenna 4	6.25	−4.9	−14.1	11.1	10
Proposed antenna	6.46	−11.25	−15.8	17.7	10

**Table 3:** Performance comparison with previous studies.

Ref	Freq. (GHz)	Type	Gain Increase (dB)	Back Radiation Reduction (dB)	FBR (dB)	Distance (λ_0_)
[9]	2.4	HIS	1.3	N/A	15	0.16
[23]	2.44	AMC	5.17	N/A	5	0.07
[24]	4.45	AMC	8	15	20	0.4
[25]	0.45	AMC	3	10	11.6	0.012
[26]	1.6	AMC	2	7	8	0.04
This work	2.7	MM Absorber	2.76	15	17.3	0.09
